# Prioritising ‘already-scarce’ intensive care unit resources in the midst of COVID-19: a call for regional triage committees in South Africa

**DOI:** 10.1186/s12910-021-00596-5

**Published:** 2021-03-22

**Authors:** Reshania Naidoo, Kantharuben Naidoo

**Affiliations:** 1 Centre for Global Health and Tropical Medicine, Nuffield Department of Medicine, University of Oxford, Oxford, UK; 2 Department of Family Medicine, School of Nursing and Public Health, University of KwaZulu-Natal, KwaZulu-Natal, South Africa

**Keywords:** Critical care triage, Critical care South Africa, COVID-19 triage, ICU, Intensive care, Rationing, COVID-19, Pandemic, Critical care, Ventilation, Scarce resources, Ethics, Governance

## Abstract

**Background:**

The worsening COVID-19 pandemic in South Africa poses multiple challenges for clinical decision making in the context of already-scarce ICU resources. Data from national government and the last published national audit of ICU resources indicate gross shortages. While the Critical Care Society of Southern Africa (CCSSA) guidelines provide a comprehensive guideline for triage in the face of overwhelmed ICU resources, such decisions present massive ethical and moral dilemmas for triage teams. It is therefore important for the health system to provide clinicians and critical care facilities with as much support and resources as possible in the face of impending pandemic demand. Following a discussion of the ethical considerations and potential challenges in applying the CCSSA guidelines, the authors propose a framework for regional triage committees adapted to the South African context.

**Discussion:**

Beyond the national CCSSA guidelines, the clinician has many additional ethical and clinical considerations. No single ethical approach to decision-making is sufficient, instead one which considers multiple contextual factors is necessary. Scores such as the Clinical Frailty Score and Sequential Organ Failure Assessment are of limited use in patients with COVID-19. Furthermore, the clinician is fully justified in withdrawing ICU care based on medical futility decisions and to reallocate this resource to a patient with a better prognosis. However, these decisions bear heavy emotional and moral burden compounded by the volume of clinical work and a fear of litigation.

**Conclusion:**

We propose the formation of Provincial multi-disciplinary Critical Care Triage Committees to alleviate the emotional, moral and legal burden on individual ICU teams and co-ordinate inter-facility collaboration using an adapted framework. The committee would provide an impartial, broader and ethically-sound viewpoint which has time to consider broader contextual factors such as adjusting rationing criteria according to different levels of pandemic demand and the latest clinical evidence. Their functioning will be strengthened by direct feedback to national level and accountability to a national monitoring committee. The potential applications of these committees are far-reaching and have the potential to enable a more effective COVID-19 health systems response in South Africa.

## Background

The novel COVID-19 outbreak was declared a pandemic by the World Health Organization (WHO) on 11 March 2020 [1]. Since this declaration, the pandemic has spread rapidly across continents with 9,296,202 cases and 479,133 deaths globally according to the WHO Situation Report dated 25 June 2020 [2]. Currently, Africa makes up 2.6% (248,558) of these cases, with South Africa making up a third of all cases in Africa [3]. With the number of COVID-19 cases in South Africa rising rapidly since easing of lockdown measures, the epicentre of the pandemic could soon shift to the African continent.

Found at regional and tertiary care level, ICU’s are mainly defined by the presence of suitably trained nurses and doctors and sophisticated medical equipment, including mechanical ventilators and other advanced monitoring physiological devices that enable potential reversal of organ dysfunction. Triage, from the French word “trier”, means “to sort”. The practice of triage is utilitarian in nature and aims to bring “the greatest good to the greatest number of people” [4]. This practice was commonplace in the battlefield where rapid decisions had to be made to optimise the utilisation of extremely scarce resources. Critical care triage during a pandemic, against the background of historical resource-limitations, is clouded by ethical complexity and as a result is emotionally and psychologically challenging for the triage officer, who is likely to be the sole treating clinician in the South African context.

The application of triage guidelines, beyond medical criteria, has proven to be highly subjective, dependent on the personal bias of and the level of seniority of the deciding clinician [5]. Triage officers will likely encounter numerous ethical dilemmas, such as which patient ought to get ‘the last ICU bed’, which patient to prioritise among those patients who score equally according to triage criteria, when to withdraw care based on medical futility and reallocation of ICU resources, all while trying to navigate and accommodate patients’ and families’ treatment preferences.

### Impact of COVID-19 on existing healthcare systems

A cross-sectional study of all China’s COVID-19 cases reported until 11 February, 2020 found that approximately 15% of patients develop severe disease requiring hospitalisation and high-flow nasal cannula oxygen support and a further 5% develop critical disease with complications such as hypoxaemic respiratory failure, acute respiratory distress syndrome(ARDS) and multiorgan failure, requiring ICU admission for mechanical ventilation [6, 7]. In Lombardy, Italy’s hardest hit region, up to 16% of all infected cases required ICU admission during the peak of the epidemic until March 7, 2020 [8]. Most recently a New York study, consisting of a cohort of 40,000 hospitalised patients with COVID-19, found that 22% of patients had hypoxaemic respiratory failure, and approximately 79% of patients required invasive mechanical ventilation [9]. Applying this to the South African context, limited ICU resources will be further subject to pressure in the form of patients presenting with various other medical or surgical conditions requiring ICU and ventilator support, including patients with AIDS-defining conditions.

Throughout Africa, the COVID-19 epidemic is occurring against a background of porous borders, weak health care systems and their failing infrastructure, inadequate surveillance and laboratory facilities, health worker constraints, personal protective equipment (PPE) shortages, vast areas of informal housing and limited budgets [10]. More specifically, South Africa bears the double burden of HIV and TB infectious disease epidemics with 7.7 million people living with HIV/AIDS and an incidence of 301,000 TB cases per year according to 2018 estimates [11, 12]. Despite South Africa having the largest ARV roll-out in the world, poor adherence to antiretroviral and TB treatments make these epidemic diseases already difficult to contain.

### Intensive care unit capacity in South Africa

**Fig. 1:**
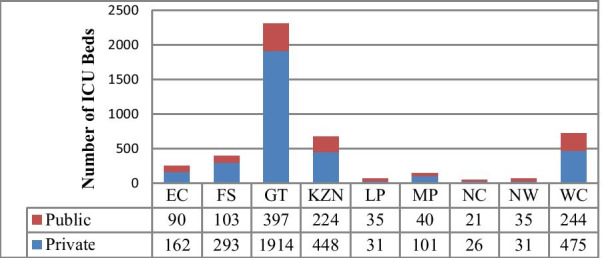
Total private and public ICU/high care beds in South Africa 2009. *EC* Eastern Cape, *FS* Free State, *GT* Gauteng, *KZN* KwaZulu-Natal, *LP* Limpopo, *MP* Mpumalanga, *NC* Northern Cape, *NW* North West, *WC* Western Cape

Independent modelling by the South African COVID-19 Modelling Consortium has projected a need for 20,000–35,000 ICU beds between June and November 2020, and that ICU capacity could be overwhelmed by July 2020 [13]. Estimates of the number of ICU beds in the country have varied widely due to a lack of research. Differing sources estimate the current number of ICU beds nationally to range from 3300 to 7000 beds. The South African Department of Health has disclosed varying estimates of ICU beds over the past few months attributed to confusion over their definitions of high-care, critical care and ICU beds [14, 15]. The last national audit of ICU resources in South Africa (2009) indicated that there were 4719 ICU and high-care beds in the private and public sectors in South Africa to cater for a population of 57 million people. Of the total number of ICU/high-care beds, 75% (3533) were in the private sector and 25% (1186) were in the public sector which alone caters for 84% of the population [16] . The majority of ICU beds in the public sector were located in three provinces (Fig. 1): Gauteng (49%, 2312 beds), KwaZulu-Natal (14%, 672 beds) and the Western Cape (15%, 719 beds) [16]. Although private facilities may be used by the State in a national emergency, there is yet to be an official national prioritisation plan set out by the South African government to enable this [17] the gross shortage of certified clinicians, coupled with the fact that only 25.6% of ICU nurses are ICU-trained, further limits the provision of intensive care [18, 19].

The rapidly widening gap between intensive care resource availability and demand for these resources begs the following ethical and moral question: How can scarce ICU resources be allocated fairly during the COVID-19 pandemic? While the Critical Care Society of Southern Africa (CCSSA) provides a guideline for the allocation of intensive care resources during the COVID-19 Public Health Emergency [20], this article highlights important considerations for clinical triage decision-making and outlines a pragmatic triage approach through formation of regional triage committees. Amidst the impending pressures and moral contentions that the pandemic presents, triage teams will be called upon to decide between improving public health outcomes and prioritising the health of the individual patient. Hence, it is even more important for clinicians and triage teams to receive support in negotiating ethical and clinical considerations underlying decision-making in the ICU.

## Main text

### Current local guidance: Critical Care Society of Southern Africa Guidelines

Evidence-based guidelines, informed by professional consensus and validated scientific-evidence, are important in reducing the burden of decision-making on individual practitioners and should ideally be subjected to periodic review and revision incorporating public input or new evidence [21]. Based on guidance from the University of Pittsburgh, USA [22], the Critical Care Society of Southern Africa (CCSSA) published guidelines on the allocation of scarce resources during the COVID-19 public health emergency in South Africa in anticipation of the increased demand for ICU bed capacity [20]. The guidelines were adopted rapidly from the University of Pittsburgh guidelines as a matter of urgency, given how quickly the pandemic was developing and have been subject to one minor revision since (D Gopalan—CCSSA President, 2020, personal communication, 23 November). These are currently the only available COVID-19 critical care guidelines published by an African critical care body and their suitability has been the subject of debate [23, 24]. The guidelines mention three main components: the formation of triage decision teams, the allocation framework for ICU resources and reassessment criteria for ongoing or withdrawing care.

Formation of triage teams, including an acute care physician triage officer and others if resources allow, will be responsible for decisions made on allocation of scarce resources using the allocation framework. These teams will also be involved in family appeals against triage decisions. The overall purpose of these teams is to minimize ‘moral distress’ of treating clinicians and allow objective decisions to be made. Given the current human workforce constraints in ICU’s, the real-life applicability of these triage teams will be difficult. An important suggestion is made for triage teams to include an ethicist and a public representative to support their ICU’s in making these decisions. As suggested later in the article, rapid formation of regional pandemic triage support teams could be instrumental in lending virtual support to under resourced ICU’s as is the current practice in France [25].

The CCSSA allocation framework makes use of population utility ethical principles to maximise optimal outcomes for as many people as possible. If the patient is deemed to be critically ill enough to require ICU care (i.e. requiring mechanical ventilator or organ support only available in ICU) and there are no other contraindications to ICU care (such as conditions regarded as medically futile like multi-organ failure or advance directives) then initial assessment is made using the Clinical Frailty Scale (CFS) based on the functioning of the patient prior to presentation. A CFS of < 6 indicates qualification for ICU care. The combined priority score is then assigned to the patient based on the sum of long-term survival prognosis (via medical assessment of comorbid conditions) and short-term survival prognosis (via Sequential Organ Failure Assessment—SOFA score, based on clinical and laboratory data). This priority score is further converted into colour-coded priority groups to facilitate ease of implementation in the hospital setting and will be applicable when available ICU resources are limited. These priority scores are subject to review by triage teams on a daily or twice-daily basis.

It is safe to assume that there will be situations where multiple individuals fall into the same priority groups. This will be a clinical and morally challenging decision for both clinicians and triage teams to make and requires careful consideration that goes beyond utilitarian considerations alone. According to the CCSSA guidelines, these ties are to be ranked in the following order: life-cycle considerations, individuals who perform tasks vital to the public health response and actual raw priority score consideration. Life cycle considerations are made on the ethical basis of providing the young an opportunity to live through life’s stages [26]. Those who perform tasks vital to the public health response are to be prioritised based on their ‘instrumental value’ and their role in overcoming the pandemic [26].

### The ethical basis for rationing scarce ICU resources

Bioethical principles provide a useful framework to assist the clinician in making decisions regarding the allocation of scarce resources. The principlist approach, defined by Beauchamp and Childress [27], consists of four key ethical principles in reflecting on moral problems towards reaching an ethical resolution: autonomy (self-governance, respect for the patient and supporting autonomous decisions), beneficence (promoting benefit), distributive justice (fair distribution of benefits and burdens) and non-maleficence (avoiding harm). Each of these four principles ought to be weighed equally and are generally considered non-hierarchical, however, they may sometimes conflict.

During a pandemic, triage and resource-allocation requires balancing of the individual patient’s rights, (underlined by the principlist approach) with that of protecting the health of the broader population. Therefore, a utilitarian approach (a form of consequentialist ethics) becomes increasingly relevant during a pandemic because the most efficient use of resources would minimise mortality and disability in the population as a whole (Table 1).

**Table 1:** Ethical theories applicable to the allocation of scarce ICU resources during the COVID-19 pandemic

Ethical principle	Applicability to COVID-19 pandemic scenario
Utilitarianism	Patients receiving priority for admission to ICU based on prognostic scoring, life-cycle considerations, societal value
Maximize overall benefits for the maximum number of people	Contention: sacrifice of duty of care to individual to maximise public utility
Egalitarianism	Equal treatment of competing patients by allowing rationing based on first-come, first-served basis or random selection (horizontal equity)
Eliminating inequality and equality as the overall goal	Consideration be given first to those with greatest need (vertical equity)
	Contention: Conflict with current triaging frameworks, potential to deprive others who may benefit
Libertarianism	Patients can access private health care if they have the means to do so
Resource distribution according to market principles	
Communitarianism	Sharing of frameworks with greater society and ensuring public support and understanding of triage policies
Respect for communities, societal order and what binds them	

It is important to note that there is no prioritisation of one ethical approach over another. Emanuel et al. [26] reiterate this point by stating that no single ethical approach in itself is sufficient in making allocation decisions in the time of COVID-19. Instead, they suggest a ‘multivalue ethical framework’ tailored to the given context. Within South Africa, application and consideration of the various ethical approaches in critical care decision-making during an evolving pandemic remains difficult due to lack of expertise and support.

The South African legal system standpoint on scarce-resource allocation can be inferred from the case of *Soobramoney v Minister of Health, KwaZulu Natal *[28]. Mr. Soobramoney was denied dialysis at a public hospital for treatment of end-stage chronic renal failure. Dialysis was denied based on not meeting the eligibility criteria of the Dialysis Unit, which was to dialyse those who had a reasonable prospect of reversibility of organ dysfunction and were eligible for kidney transplants. These criteria were in place due to the hospital having only eight dialysis beds. Mr Soobramoney argued that this decision was an infringement of his right to life and access to emergency health care in terms of The Constitution of the Republic of South Africa (Act No.108 of 1996) [29]. The Constitutional Court ruled in favour of the hospital, given that with its limited dialysis resources it would only be able to provide dialysis to patients with certain clinical profiles. In *Soobramoney v Minister of Health, KwaZulu-Natal,* Sachs J observed that:

In all the open and democratic societies based upon dignity, freedom and equality with which I am familiar, the rationing of access to life-prolonging resources is regarded as integral to, rather than incompatible with human rights approach to health care. [28]

### Moving beyond scarce allocation frameworks: important considerations in triage decision-making

#### Pitfalls of CFS and SOFA

Utility of the CFS and SOFA are not without their flaws. Pugh et al. [30], in their assessment of inter-rater reliability of the CFS in a prospective observational study found that factors independently associated with higher frailty ratings were: female sex; higher Acute Physiology and Chronic Health Evaluation II score; higher category of pre-hospital dependence; and the assessor having a medical background, with score differences of at least 1 point in 47% of cases. Singh and Moodley [23] further critique use of the CFS in that assessment of function 1–2 weeks prior to admission may not be feasible. In recognition of its pitfalls, the United Kingdom National Institute for Health and Care Excellence(NICE) Covid-19 Rapid Guideline has been updated to emphasise use of the CFS as part of a ‘holistic assessment’ and awareness of its limitations as the only assessment of frailty [31]. The guidelines advise against its use in younger people, people with stable-long-term disabilities, learning disabilities or autism.

With regards to SOFA, the laboratory clinical results on which the score is based may take time, especially in the resource-limited context of a district or rural hospital [23]. Several reports based on the outbreak in China, indicate that a smaller proportion of patients develop other organ dysfunction prior to developing respiratory failure, thus limiting the usefulness of traditional physiological measures such as SOFA and National Early Warning Scores [32]. Until better prognostic tools are available, the clinician ought to bear in mind the limitations of both the CFS and SOFA scores.

### Withdrawal of ventilatory support based on medical futility

Withdrawal of ventilatory support due to medical futility precede most deaths in the ICU and is a reality that the clinician has become accustomed to dealing with in the pre-pandemic period in South Africa [33]. However, some trepidation regarding this decision still holds true for less experienced clinical staff, especially during periods where more junior staff have to step up due to staffing need. In an ideal setting, this medical futility decision should ideally involve the senior critical care specialist, the critical care nurse, ideally an ethicist (if available) and a representative of the patients family in making the decision to withdraw further ICU care and ventilator support. This should be followed by transfer to a high-care ward, monitored by specialised palliative care staff. Phua et al. [34] found COVID-19 mortality in ICU to be associated with older age, comorbidities (including hypertension, diabetes, cardiovascular disease, chronic lung disease and cancer), higher severity of illness scores, worsening respiratory failure, higher d-dimer and CRP concentrations, lower lymphocyte counts and secondary infections. Medical futility and rationing decisions should account for these factors (and their changing levels of evidence) in the absence of formalised prognostic tools and biomarkers.

The Health Professions Council of South Africa (HPCSA) supports practitioners in their decision to withdraw treatment, stating that it is permissible to withhold treatment “even if it is not in the best interest of the patient” [35]. The World Medical Association (WMA) supports this in stating that a physician can determine that a treatment is medically futile or non-beneficial because it offers no reasonable hope of recovery or improvement or because the patient is permanently unable to experience any benefit [36]. They further state that a physician is under no obligation to provide ‘futile or non-beneficial treatment’ [36].

### Withdrawal and reallocation of ventilator support

When ICU capacity is overwhelmed, it would be ethically justifiable to withdraw care from one patient to reallocate it to another with better prognosis. This is justifiable based on population utility in the context of a public health emergency, in that population health outcomes ought to be maximised and that allowing patients indefinite use of ICU resources would go against this. Given that the decision to withdraw would need to be made quite timeously in the pandemic scenario, White et al. [37] have put forward useful recommendations for the ICU team to effectively manage this situation, which has been adapted here:

1. When the decision is made to admit a patient to ICU for mechanical ventilation, sensitively manage expectations of the family and patient adequately by presenting it as a ‘time-limited therapeutic trial’, not an unlimited resource
2. ICU management should have agreed-upon durations for the trial of ventilation. The aim should be to avoid ‘rapid cycling’ of ventilators where ventilator care is withdrawn too early in those who may have benefited if ventilated for a few more days.
3. These decisions need to be made by the triage team or officers, not the treating clinician, to ensure objectivity and minimise moral distress
4. Once the decision is made to withdraw ventilator support this should be followed by full palliation and emotional support for the family

The CCCSA guidelines have been unclear on withdrawal of ventilator support and reallocation. However, the British Medical Association acknowledges that in the context of overwhelming demand, withdrawal of lifesaving treatment may be justifiable to provide life-saving treatment to a patient with better prognosis.

### A call for provincial triage support committees

The ethical and clinical triage considerations outlined can be incredibly difficult for clinicians to navigate in the face of pandemic pressures. Multi-disciplinary support committees can be invaluable in reducing the angst experienced by clinicians on the ground when making such triage decisions and can free up capacity to focus on clinical and duty-of-care responsibilities. Such committees ideally provide an impartial, broader viewpoint which will consider broader contextual factors such as adjusting rationing criteria according to different levels of pandemic demand and evolving clinical evidence [33]. Regional ethical support committees have been implemented with some success in France during the first wave of the COVID-19 pandemic, providing rapid clinical decision-making support not only to ICU’s, but GP’s and emergency medicine staff as well [25]. Formation of such committees allow the legal responsibility for such decisions to be extended from the treating clinicians to the committees themselves. Sharing of legal responsibility could address the widely-held fears around prosecution by many health care professionals, thereby allowing them ‘to make clinical decisions free from the fear of the risk of criminal charges.’ [38].

The CHEST Implementation Guide for Triage [39] suggests that triage criteria form only one part of what should be a three-component system for effective triage. The other two components would be a concept of operations (i.e., decision-making process that may include an incident command system supported by a multidisciplinary clinical care committee) and coordination with other hospitals, regional policies, and leadership to ensure uniformity. Within the LMIC setting and more specifically South Africa, there are practical constraints in achieving all components. Although the CCSSA has published detailed guidelines on triage within the ICU setting and proposed a formation of hospital triage committees, we realise that support for such a committee in each hospital may not be feasible in the LMIC setting due to staff and resource constraints. The current lack of a consistent concept of operations, cohesive policies and resource-sharing between health facilities, currently hinders effective coordination between them. Furthermore, effective information systems that communicate across healthcare institutions and provide real-time updates on bed capacity are not yet widely available in South African hospitals.

### Provincial triage committees: an adapted version for South Africa

**Fig. 2:**
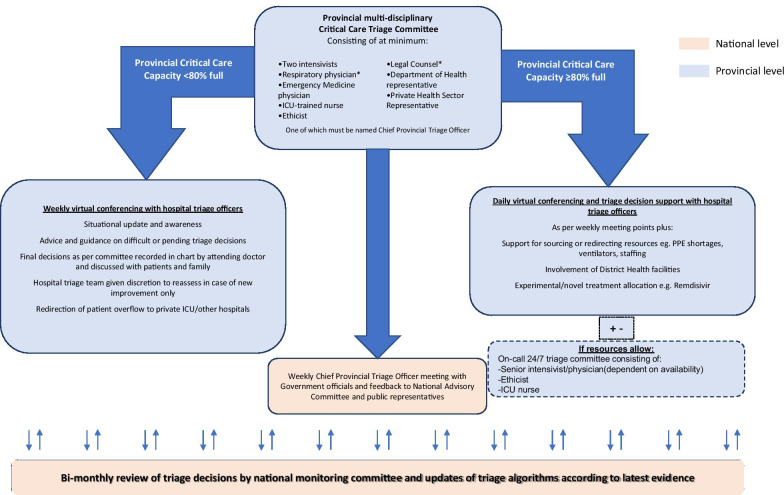
Proposed model for provincial critical care triage committees in South Africa

Effective triage, whether at the critical care or district level is heavily dependent on effective communication mechanisms between administrative staff, health-facilities, patients and their families. The authors therefore propose the below adapted provincial virtual triage critical care support mechanism for South Africa (Fig. 2), taking into account the above-mentioned constraints. This mechanism has the potential to ensure better coordination between all provincial health facilities in terms of resource-, knowledge- and capacity-sharing. Depending on the scenario—whether surge capacity is needed, or capacity is at ‘pre-pandemic levels’, will dictate the frequency and level of meetings. We have suggested an arbitrary cut-off threshold of 80% bed capacity for frequency of meetings. If resources allow (which would be province-dependent) a 24-h triage support line should be made available. The proposed triage committee structure and meeting thresholds will need to be adjusted with input from government health officials and clinicians and policies created to support its effective functioning and legal status in triage decision-making.

The overall aim of provincial triage committees will be to provide guidance and multi-disciplinary input, thereby allowing hospitals to share the patient load, discuss difficult cases, enable transfers, outsource to the private sector, and ensure region-wide consistency in decision-making. Ultimately, the burden of responsibility in the case of rationing ventilators would then fall on the provincial committee rather than the individual clinician. The authors also envision inviting district health facilities to participate as they often manage critically ill patients on the peripheries when regional ICU’s are full. The committees, through bidirectional communication, will provide a useful platform for situational awareness and rapid assessments of supply and demand, given that the information systems in South Africa are not advanced enough to do so otherwise. The provincial chief triage officers will then report back to the national advisory committee on a weekly basis.

As per recommendations made by Daugherty et al. [40], a national monitoring committee will ensure consistency in decision-making with two-way feedback between provincial committees and national level. Centralised, impartial monitoring will allow for update of triage algorithms using the latest available evidence and ensuring governance in decision-making. Centralised monitoring will also ensure that bias due to disabilities and advanced age and other systemic inequities are not predominating triage decisions, as has been alleged in the USA [41]. Lastly, national policy supporting sharing of resources between the private and public sector would need to be solidified. With the new National Health Insurance (NHI) healthcare model transition underway in South Africa, we foresee such committees complementing the shift of critical care services under the new NHI model.

Patients deemed by the committee to be unsuitable for ventilation or for withdrawal of ventilation are to be palliated as per the Association of Palliative Care Practitioners of SA guidelines [42], with decisions clearly and compassionately communicated to families and documented. Patient family appeals will be dealt with via an appeals mechanism which will involve both the hospital triage officer and provincial triage committee. Public representatives should be incorporated into the national meetings as a means of keeping the public informed in terms of social accountability.

## Conclusion

As the South African government plans to ease lockdown measures, the critical care community waits with trepidation to see if there will be a corresponding rise in COVID-19 -infected patients presenting to hospital and requiring ventilation similar to the proportions seen in the USA and Italy. With no clear plan for ramped up ventilator-production or private facility buy-in, amongst other local failures to contain the outbreak, ICU’s are likely to become overwhelmed and not every patient deserving of ventilator support in ICU will receive such care. The CCSSA has issued valuable guidance for the allocation of ventilators based on USA guidance, utilising CFS and SOFA scores to form a raw priority score. Should there be a tie, then life cycle considerations, essential workers vital to the pandemic response and actual raw priority score should be respectively considered. CFS and SOFA scores are the best tools available until better prognostic tools and biomarkers are developed but should be interpreted with caution. Withdrawing care owing to medical futility and/or reallocation of an ICU bed to another patient is ethically defensible and supported by the HPCSA and the World Medical Association. In such cases, it is imperative to involve the family from the time of admission to ICU and to establish local policies on duration of ventilation-trials before any decisions to withdraw can be made. Above all, despite pressures imbued by the pandemic situation, the duty of care to the patient should always extend to good communication with the family and managing their expectations from early on.

In addition to severe resource constraints, we would expect critical care workers to be subjected to immense physical and emotional pressure during the pandemic period. It is essential that triage officers and teams be identified to relieve treating clinicians of the distress of making such decisions and to minimise bias where possible. The authors recommend the formation of provincial multi-disciplinary triage support committees to provide virtual support meetings to facility-based triage teams according to the model described. Resource-permitting, a 24-h support hotline should also be made available. While no single ethical approach is sufficient to guide resource allocation during the pandemic, the formation of such committees will allow for expert ethics advice and the adaptation of multivalue ethical frameworks to the evolving pandemic context. Given the unique challenges of the under resourced healthcare system in South Africa, virtual triage support committees will allow for multi-disciplinary collaboration, resource and knowledge-sharing at a vital time and will alleviate the moral and legal burden on individuals based at ICU facilities.

## Data Availability

The datasets used and/or analysed during the current article are available from the corresponding author on reasonable request.

## Abbreviations

ARDS: Acute respiratory distress syndrome
CCSSA: Critical Care Society of Southern Africa
CFS: Clinical Frailty Score
GP: General practitioner
ICU: Intensive care unit
LMIC: Low and middle income countries
NHI: National Health Insurance
NICE: National Institute for Health and Care Excellence
SOFA: Sequential Organ Failure Assessment
USA: United States of America
WHO: World Health Organisation
